# Structure basis for sugar specificity of gustatory receptors in insects

**DOI:** 10.1038/s41421-024-00716-6

**Published:** 2024-08-06

**Authors:** Ruizhu Chen, Ran Zhang, Lu Li, Bozhan Wang, Zhiwei Gao, Fenglian Liu, Yan Chen, Yutao Tian, Baobin Li, Qingfeng Chen

**Affiliations:** 1 Center for Life Sciences, Yunnan Key Laboratory of Cell Metabolism and Diseases, State Key Laboratory for Conservation and Utilization of Bio-Resources in Yunnan, School of Life Sciences, Yunnan University, Kunming, Yunnan China; 2 Department of Anesthesiology, Zhongshan Hospital, Institute for Translational Brain Research, State Key Laboratory of Medical Neurobiology, MOE Frontiers Center for Brain Science, Fudan University, Shanghai, China; 3 Academy of Medical Engineering and Translational Medicine (AMT) & Tianjin Key Laboratory of Brain Science and Neural Engineering, Tianjin University, Tianjin, China

**Keywords:** Cryoelectron microscopy, Ion channel signalling

Dear Editor,

Taste perception is essential for food selection and animal feeding behaviors^1^. While mammals use G protein-coupled receptors (GPCRs) for sweet and bitter tastant perception, insects such as fruit flies use a unique family of proteins called gustatory receptors (Grs)^2^. Attractive sweet compounds and aversive bitter compounds are perceived by different subsets of Grs distributed in distinct subpopulations of sensory cells, which eventually drive innate attraction or repellency^1^. Like mammals, this is crucial for choosing the appropriate food, and thereby for the survival of insects^1^. 8 members (Gr5a, Gr61a, and Gr64a–f) out of 68 Grs from *Drosophila melanogaster*^3,4^ are found to be located in sweet taste neurons^5^, and most of them are involved in sweet perception^6^. Among them, Gr5a, essential for trehalose sensing, is the first member with known sugar specificity^7^, whereas Gr64a was suggested to be essential for sensing multiple sugars, including glucose, sucrose, and maltose^8^. More recently, Gr43a and its homologs were identified as narrowly tuned fructose receptors, and they were also found to be expressed in the brain and function as a sensor for the hemolymph fructose levels, thereby regulating the feeding behaviors^9^.

To unravel the overall structure of these receptors and their unique structure features that define sugar specificity, we determined the cryo-electron microscopy (cryo-EM) structures of *Drosophila mojavensis* Gr43a (DmoGr43a) (given its better biochemical behavior than *Drosophila melanogaster* Gr43a (DmGr43a)) and *Drosophila melanogaster* Gr64a (DmGr64a) in apo or sugar-bound states. Despite of limited in vivo relevance, we performed electrophysiology and/or calcium imaging in artificial system (HEK293 cells) to obtain some hints about their function. These assays demonstrated that both DmoGr43a and DmGr64a can form functional ion channels (Fig. 1a–d; Supplementary Figs. S1 and S2). Among different sugars tested, e.g., 100 mM maltose, arabinose, glucose, trehalose, and fructose, only fructose activated DmoGr43a, with a measured EC_50_ of 42.97 ± 4.69 mM and ~30 mM in electrophysiology and calcium imaging respectively (Fig. 1a, b; Supplementary Fig. S1). For DmGr64a, among tested sugars, several sugars including fructose, trehalose, sucrose and maltose were able to activate DmGr64a in our calcium imaging experiments, whereas glucose and arabinose could not (Fig. 1c, d; Supplementary Fig. S2). The measured EC_50_ of sucrose in DmGr64a is ~1.77 mM.

**Fig. 1:**
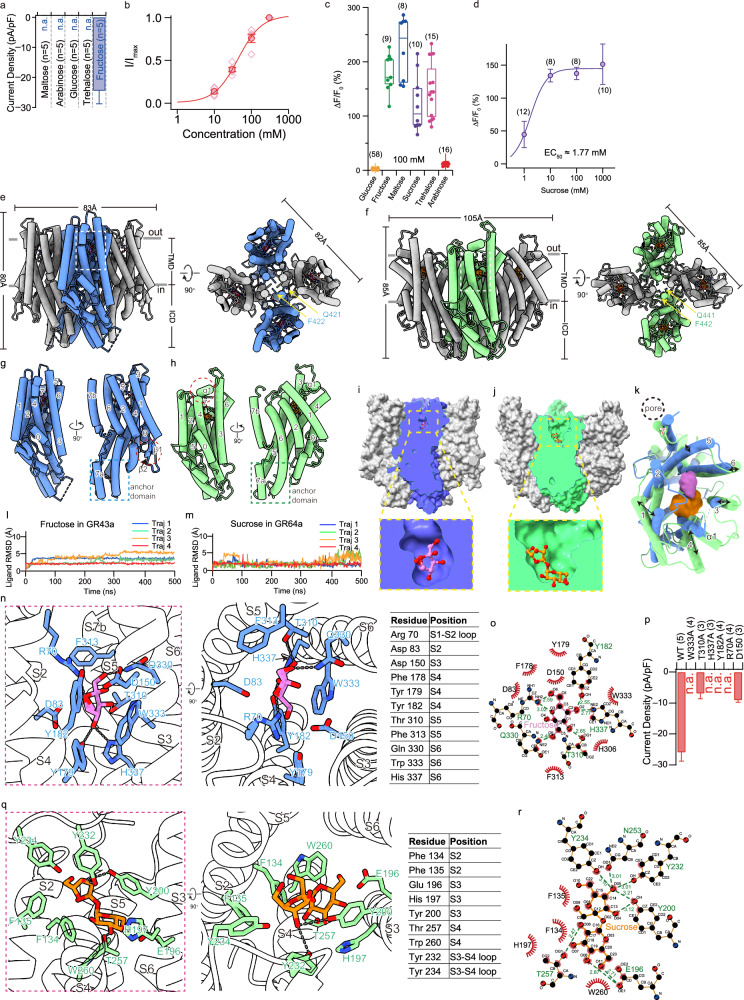
Functional and structural insight into sugar specificity of DmoGr43a and DmGr64a. **a** Current densities of DmoGr43a-expressing HEK293 cells activated by different sugars. **b** Concentration-response curve of DmoGr43a activation by fructose as measured by electrophysiology. **c** Summary of fluorescence intensity changes in DmGr64a-expressing HEK293 cells induced by various sugars. **d** Concentration-response relationship of sucrose and DmGr64a. **e**, **f** Cartoon representation of fructose-bound DmoGr43a (**e**) and sucrose-bound DmGr64a (**f**) structures in two views (front and top). **g**, **h** Cartoon represents a single subunit of fructose-bound DmoGr43a structure (**g**) and sucrose-bound DmGr64a structure (**h**) in two views. **i**, **j** Surface representation of fructose-bound DmoGr43a (**i**) and sucrose-bound DmGr64a (**j**) structures in front views, with the foremost subunit sliced for clarity. **k** Overlay of single subunit of fructose-bound DmoGr43a and sucrose-bound DmGr64a structures viewed from above, highlighting the splaying of some TM helices at the extracellular half. **l**, **m** The time-dependent RMSD of fructose and sucrose concerning their starting coordinates during the 500-ns simulation. RMSDs of 4 bound sugars in each tetrameric channel are overlaid. **n** Interactions between fructose and DmoGr43a in two views, with the former being an expanded view of the region boxed in **e**. The table on the right lists residues involved in fructose binding. **o** Schematic representation of the interactions between fructose and DmoGr43a as calculated by LIGPLOT. **p** Current densities for various DmoGr43a mutants evoked by 100 mM fructose. **q** Interactions between sucrose and DmGr64a in two views, with the former being an expanded view of the region boxed in **f**. The table on the right lists residues involved in fructose binding. **r** Schematic representation of the interactions between sucrose and DmGr64a as calculated by LIGPLOT. Fructose and sucrose are colored violet and orange respectively throughout the figure. Data are presented as mean ± SEM in **a**–**d**, **p**. The measurements were conducted in multiple independent cells (*n* in **a**, **c**, **d**, **p** as labeled and *n* = 5 in **b**).

Both DmoGr43a and DmGr64a were expressed and purified to homogeneity for single particle cryo-EM analysis, yielding 4 structures in distinct states: DmoGr43a apo state, DmoGr43a fructose-bound state, DmGr64a apo state and DmGr64a sucrose-bound state (Fig. 1e–h; Supplementary Figs. S3–S6). As expected, both DmoGr43a and DmGr64a form tetramer, and are topologically similar to each other, and to Orco from *Apocrypta bakeri*^10^ and odorant receptor 5 from *Machilis hrabei* (MhOR5)^11^ (Fig. 1; Supplementary Figs. S7–S8). Structure of DmGr64a is more expanded than that of DmoGr43a, particularly in the transmembrane domain (TMD, Fig. 1), which appears to partially account for the differences in their sugar specificity. The structures of DmoGr43a and DmGr64a can be separated into a TMD with 28 transmembranes (TMs) in total (7 TMs in each subunit, numbered as S1–S7), and an intracellular domain (ICD) containing an “anchor domain”, following the nomenclature of Orco/MhOR5^10,11^ (Fig. 1g, h). Inter-subunit interfaces in TMD are formed exclusively by S7b, leaving large cavities between the neighboring subunits (lipid inlets), which are filled with large number of lipids in both proteins (Supplementary Fig. S8). Intriguingly, one lipid molecule is bound stably in each inter-subunit interface and penetrates the ion conduction pore (Supplementary Fig. S9), and may play a role in channel assembly or activation.

Four sugar binding pockets are observed in DmoGr43a and DmGr64a tetramer, one in each subunit, positioned similarly to odor molecule-binding sites in Orco/MhOR5 (Fig. 1i, j; Supplementary Fig. S10b)^10,11^. Yet, the size and chemical environment of ligand binding sites are drastically different between Grs and ORs, correlating with the distinct chemical nature of their ligands (hydrophilic, generally larger sugar molecules vs hydrophobic, smaller odor molecules). While the binding sites in Grs are solvent-exposed and larger in size, binding sites in MhOR5 are solvent inaccessible and smaller in size (Fig. 1i, j; Supplementary Fig. S10b). The sugar binding pocket is surrounded by S1–S6 in the TMD of each subunit in both proteins, with the one for DmGr64a being much larger (Fig. 1i, j), consistent with a wider sugar spectrum observed in DmGr64a. Interestingly, Gr5a and other Grs in Gr64a–f also appear to have larger ligand binding pockets, as demonstrated by comparing their AlphaFold models with DmoGr43a structure (Supplementary Fig. S11). Larger sugar binding pocket in DmGr64a results from splaying of extracellular half of the helical bundle (S1–S6) that harbors the binding site (Fig. 1k). This structural difference appears to partially account for the expanded TMD in Gr64a (Fig. 1e, f, k). Among these helices, the extracellular halves of S1, S3, and S4 undergo the most dramatic outward tilting in DmGr64a when compared with DmoGr43a (Fig. 1k). When the protomer structures of sugar-bound DmoGr43a and DmGr64a were aligned, it shows that these two sugar molecules do not overlap with each other, with the bound fructose molecules positioned closer to the pore in DmoGr43a, and the bound sucrose molecules in DmGr64a positioned further away from the pore (Fig. 1k).

Fructose, a pentose, adopts a conformation that its five-membered ring is roughly perpendicular to the membrane surface in the fructose-bound DmoGr43a structure (Fig. 1i). Accurate modeling of bound fructose in DmoGr43a was confirmed using molecular dynamic (MD) simulations, where fructose molecules remained stable (Fig. 1l; Supplementary Fig. S12). When glucose was placed in the densities, they also have a high probability of stable binding in the pocket according to the MD simulations (Supplementary Fig. S13), in line with the observation that glucose could bind but not activate BmGr9^12^. Binding of fructose in DmoGr43a is achieved by formation of hydrogen bonds and hydrophobic interactions between residues in the binding pocket and various groups of fructose (Fig. 1n, o). Notably, Arg 70 from the S1–S2 loop, which is disordered in the apo state, becomes well-resolved in the fructose-bound state, and travels downward for fructose binding (Supplementary Figs. S14 and S15). Residues participating in fructose binding are generally very sensitive to mutagenesis, as most mutations rendered the channel non-responsive to fructose at up to 300 mM concentration (Fig. 1p). In these mutants, protein expression and localization are not significantly affected (Supplementary Fig. S16).

Sucrose, a disaccharide that is a conjugate of glucose and fructose, has an overall elongated shape, and inserts deeply into the sugar binding pocket of DmGr64a, with the fructose moiety being closer to the entrance and the glucose moiety sitting deeper in the pocket (Fig. 1j). Accurate modeling of bound sucrose in DmGr64a was confirmed using MD simulations, where sucrose molecules remained stable (Fig. 1m; Supplementary Fig. S12). By contrast, when glucose was placed in the densities, it could not bind stably in the pocket according to MD simulations (Supplementary Fig. S13). Binding of sucrose in DmGr64a was achieved by formation of hydrophilic and hydrophobic interactions between residues in the binding pocket and various groups of sucrose (Fig. 1q, r). Similarly, in sucrose-bound state, these residues are brought closer to sucrose molecules, with Tyr 234 located in the loop between S3 and S4 being most dramatic (Supplementary Fig. S15). Despite that the measured EC_50_ for some mutants might be inaccurate due to limit in the highest concentration of sucrose used, mutation of sucrose binding residues obviously decreased its sensitivity to sucrose, as shown in the calcium imaging experiments (Supplementary Fig. S17), without significantly affecting channel expression and localization (Supplementary Fig. S18).

Lined by S7 that spans both ICD (S7a) and TMD (S7b), a continuous ion conduction pathway in the 4-fold symmetry axis runs all the way through both DmoGr43a and DmGr64a, and are all in the non-conductive state (Supplementary Fig. S19a, b, d, e). Therefore, sugar-bound structure of DmoGr43a and DmGr64a are likely in desensitized or pre-open states. In both proteins, two patches of constrictions are formed along the ion conduction pathway, one near the extracellular entrance (Ile 418 and Phe 422 for DmoGr43a, and Val 438 and Gln 441 for DmGr64a) and the other in the anchor domain in ICD (Leu 386 for DmoGr43a, and Glu 399 and Phe 403 for DmGr64a) (Supplementary Fig. S19). Intriguingly, the narrowest point at the extracellular entrance is formed by completely different sets of residues in DmoGr43a and DmGr64a, e.g., bulky, hydrophobic phenylalanine in DmoGr43a vs smaller, hydrophilic glutamine in DmGr64a (Supplementary Figs. S19 and S20). Most likely, the constrictions at the extracellular side would be released upon sugar binding, whereas the ones in ICD would remain constricted, and ions would travel through the side fenestrations located at ICD–TMD junction (Supplementary Fig. S9), as are the cases in Orco/MhOR5^10,11^.

When the apo and sugar-bound structures of DmoGr43a and DmGr64a were aligned, notable conformational changes could be revealed, mainly tilting of TM helices responsible for sugar binding towards bound sugars (Supplementary Fig. S15). In DmoGr43a, Phe 313 from S5, which is directly involved in fructose binding, moves ~1.6 Å towards fructose, together with the whole S5 (Supplementary Fig. S19c). Similarly, a bulky residue in S5 of DmGr64a, Phe 333, undergoes movement towards sucrose (Supplementary Fig. S19f). The difference is, Phe 333 in DmGr64a is not directly involved in sucrose binding. However, it forms interaction with Trp 260 in S4 that is directly involved in sucrose binding (Supplementary Fig. S19f). We speculate that, in both proteins, S5 plays an important role in channel activation by their ligands, as it is next to and tightly coupled with the pore-forming S7s. For example, hydrophobic interactions are formed between Tyr 312, Phe 315 from S5 and Leu 419, Ile 420 from S7 of DmoGr43a (Supplementary Fig. S19c), and between Leu 338, Leu 339, Phe 342 from S5 and Ile 420, Leu 439 from S7 of DmGr64a (Supplementary Fig. S19f). The local conformational changes in the ligand binding pocket will likely be propagated to S7 via S5, leading to channel opening, as have been shown recently^12,13^.

In a recently published study^13^, the same Gr64a homolog (both from *Drosophila melanogaster*) but different Gr43a homologs (*Drosophila mojavensis* vs *Drosophila melanogaster*) were used for cryo-EM structural studies, where the overall structure (RMSDs of 1.46 Å for Gr64a apo structure and 0.87 Å for Gr43a apo structure), location of sugar-binding pocket and pose of bound sugar molecules are highly similar to each other. These studies are therefore nice cross-validation of each other. Moreover, our analysis of the AlphaFold model of other Gr64a homologs provides initial insight into their diverse sugar specificity. When comparing our DmoGr43a structure with that of BmGr9 reported recently^12^, although their structural similarity is less striking (a sequence identity of ~25% and an RMSD of 2.12 Å when superimposed), they have the same sugar specificity (Supplementary Figs. S7 and S8). Taken together, the findings in this study and other recent studies^12,13^ provide a framework for understanding the mechanism of sweet perception in insects.

### Supplementary information

Supplementary information

## Data Availability

Structure coordinates and cryo-EM density maps have been deposited at the Protein Data Bank and Electron Microscopy Data Bank under accession numbers 8ZDZ and EMD-60018 for DmoGr43a in apo state; 8ZE3 and EMD-60022 for DmoGr43a fructose-bound state; 8ZE0 and EMD-60019 for DmGr64a in apo state; and 8ZE2 and EMD-60021 for DmGr64a in sucrose-bound state. Accession numbers for other structures analyzed in this manuscript are indicated in the figures and can be downloaded from the Protein Data Bank.
