# Supplementary material for: Structure basis for sugar specificity of gustatory receptors in insects
Source: Cell Discov. 2024 Aug 6;10:83. doi: 10.1038/s41421-024-00716-6 (PMC11300463; doi:10.1038/s41421-024-00716-6)
Supplement: Supplementary file 1 — Supplementary information [file 41421_2024_716_MOESM1_ESM.pdf]

Structure basis for sugar specificity of gustatory receptors in insects

Ruizhu Chen[1*], Ran Zhang[2*], Lu Li[3*], Bozhan Wang[3*], Zhiwei Gao[3], Fenglian Liu[1], Yan Chen[1], Yutao Tian[3#], Baobin Li[2#], Qingfeng Chen[1#]

[1]Center for Life Sciences, Yunnan Key Laboratory of Cell Metabolism and Diseases, State Key Laboratory for Conservation and Utilization of Bio-Resources in Yunnan, School of Life Sciences, Yunnan University, Kunming 650091 China

[2]Department of Anesthesiology, Zhongshan Hospital, Institute for Translational Brain Research, State Key Laboratory of Medical Neurobiology, MOE Frontiers Center for Brain Science, Fudan University, Shanghai 200032, China

[3]Academy of Medical Engineering and Translational Medicine (AMT) & Tianjin Key Laboratory of Brain Science and Neural Engineering, Tianjin University, Tianjin, P.R. China, 300072

* These authors contributed equally.

# Correspondence and requests for materials should be addressed to qingfengchen@ynu.edu.cn, libaobin@fudan.edu.cn, or yutao.tian@tju.edu.cn

This pdf file includes:

Materials and Methods

Supplementary Fig. S1 to S20

Supplementary Table S1

Materials and Methods

Protein expression, purification, and nanodisc reconstitution

Coding sequence of DmoGr43a (NCBI refseq XM_044011021.1) was synthesized by Tsingke ltd, whereas coding sequence of DmGR64a was amplified from total cDNA of *Drosophila melanogaster*. Coding sequences of DmoGr43a and DmGr64a were fused with a C-terminal strep tag and cloned into a pEZT vector[1], which were subsequently expressed in HEK293F cells using the BacMam system. Sf9 cells and HEK293F cells were grown in SIM SF Expression Medium (Sino Biological Inc) and SMM 293-TI complete medium (Sino Biological Inc) respectively, supplemented with 2% fetal bovine serum (FBS, Biological Industries). The baculovirus was generated in Sf9 cells following standard protocols using transfection reagent X-tremeGENE™ (Roche) and used to infect HEK293F cells at a ratio of 1:40 (virus:HEK293F, v:v).

48 hours after infection, cells were harvested by centrifugation at 4,000 g. Subsequent purification steps were carried out at 4°C. The cell pellet was resuspended in buffer A (50 mM Tris pH 8.0, 200 mM NaCl), supplemented with 1 mM PMSF and disrupted by sonication on ice. The lysate was centrifuged at 40,000 RPM for 1 h to pellet membranes, which were then Dounce homogenized in buffer A supplemented with 1 mM PMSF. DmoGr43a and DmGr64a were extracted with 1% (w:v) n-Dodecyl-beta-Maltoside (DDM) supplemented with 0.02% (w:v) Cholesteryl hemisuccinate (CHS) by gentle agitation for 2 hours. Insoluble material was removed by centrifugation at 20,000 RPM for 40 minutes and the supernatant was incubated with Strep-Tactin®XT (IBA Lifesciences) for 2 hours with gentle agitation. The resin was then collected in a disposable gravity column (Bio-Rad), washed with buffer A supplemented with 0.05% (w:v) DDM, and finally eluted with elution buffer provided by manufacturer, supplemented with 0.05% (w:v) DDM and 0.002% (w:v) CHS. For DmoGr43a, the eluant was concentrated, and further purified by size exclusion chromatography on a Superose 6 10/300 GL column (GE Heathcare) pre-equilibrated with buffer B (20mM Tris pH 8.0, 150mM NaCl) supplemented with 0.06% (w:v) GDN. The peak was collected and concentrated to ~4 mg/ml for cryo-electron microscopy analysis.

Eluant of DmGr64a during affinity purification was directly reconstituted into nanodiscs, following steps reported previously[2]. In brief, MSP1, DmGr64a, and lipid (POPC:POPG:POPE = 3:1:1) were mixed at a molar ratio of 4:1:10 respectively. After incubating on ice for 30 min, detergents were removed by adding 100 mg ml^{-1} Bio-Beads SM2 (Bio-Rad), with gentle agitation for 4 h. After repeating this step twice with fresh Bio-Beads, the sample was loaded onto a Superose 6 10/300 GL column pre-equilibrated with buffer C (20 mM HEPES pH 7.0 and 150 mM NaCl). The peak fraction containing DmGr64a reconstituted into nanodiscs was collected for cryo-EM analysis.

Grid preparation

The DmoGr43a and DmGr64a samples were centrifuged at 21,000 x g for 10 min at 4 ℃ before grids preparation. For complex preparation, different ligands were added at a final concentration of 50-100 mM to the concentrated sample (~4 mg/ml for DmoGr43a and ~1 mg/ml for DmGr64a) and incubated on ice for 30min. A 3 μL sample was applied to holey carbon, 300 mesh R1.2/1.3 gold grids (Quantifoil, Großlöbichau, Germany) that were freshly glow discharged in H2-O2 mixture for 30 seconds. Sample was incubated for 5 seconds at 4 ℃ and 100% humidity prior to blotting with Whatman #1 filter paper for 3-3.5 seconds at blot force 1 and plunge-freezed in liquid ethane cooled by liquid nitrogen using a FEI Mark IV Vitrobot (FEI / Thermo Scientific, USA).

Cryo-EM data acquisition and data processing

Grids were clipped and transferred to a Titan Krios G4 cryo-electron microscope operated at 300 kV, equipped with a Falcon G4i direct electron detector. Movie stacks were recorded using the EPU software (Thermo Fisher Scientific) in super-resolution mode with pixel size of 0.466 Å. The total dose per EER (electron event representation) movie was 49.95 e^-/Å, 48.66 e^-/Å, 49.52 e^-/Å and 48.24 e^-/Å for apo DmoGr43a, fructose-bound DmoGr43a, apo DmGr64a and sucrose-bound DmGr64a datasets, respectively.

For DmoGr43a apo dataset, 7,587 micrographs were collected and each EER movie of

1,080 frames were fractionated into 40 subgroups and corrected for beam-induced drift using MotionCor2[3] in Relion (v4.0.0)[4] and the data was binned to 0.932 Å/pixel. Then, exposure-weighted micrographs were imported into cryoSPARC (v4.0.2)[5] for contrast transfer function (CTF) estimation by patch CTF. Particles were blob-picked in 50 micrographs and extracted with a box size of 220 pixels, and subjected to 2D classification. Good classes were selected and served as the template for the topaz training or template-based picking. Then particles from all micrographs were extracted and subjected to a couple rounds of 2D classifications. After initial cleanup, classes with clearly defined and recognizable features were used to generate an ab initio model with 2 classes and 0 similarity with or without symmetry. Particles belonging to a class with well-defined features were further refined using Heterogeneous refinement and Non-uniform (NU) refinement with C4 symmetry, which finally yielded a map at 2.64 Å for model building.

For fructose-bound Gr43a, apo Gr64a, and sucrose-bound Gr64a, total of 7,965, 3,342, and 4,005 EER movie stacks were collected and processed in a similar way as Gr43a apo dataset and yielded final maps at 3.17 Å, 2.54 Å, and 2.57 Å respectively.

Model building, refinement and validation

Model building was manually conducted in Coot, using AlphaFold model deposited in Uniprot as a starting point (AF-Q9V4K2-F1 for DmoGr43a and AF-P83293-F1 for DmGr64a). Models were refined against cryo-electron microscopy maps using real-space refinement in PHENIX[6], and validated using the validation tool implemented in PHENIX to avoid overfitting. To facilitate modeling and refinement of the fructose and sucrose molecules, CIF files were generated by using eLBOW implemented in PHENIX[6]. The final models include residues 1–160, 167-216, and 264-423 for both structures of DmoGr43a, and residues 47–447 for both structures of DmGr64a. The statistic of the models' geometries was generated using MolProbity[7]. Pore radii were calculated using the HOLE program[8]. The diagram for protein and ligand interaction was generated by the LIGPLOT program[9]. All figures were prepared in PyMol[10] or ChimeraX[11].

Electrophysiology recordings

DmoGr43a and its mutants were expressed using the pCAGIG vector in HEK tsA cells. Cells were maintained in DMEM with glutamine and 4.5 g liter^{-1} glucose (Gibco) supplemented with 10% fetal bovine serum (Gibco) and penicillin–streptomycin (0.1 U liter^{-1}; Gibco) at 37 °C and 5% CO2. For each 35-mm dish, 1 μg of plasmid DNAs encoding DmoGr43a were transfected into the cells with 3 μl of GenJet.II (SignaGen) according to the vendor instructions. Mutations were generated by standard PCR-mediated mutagenesis (Takara Bio) using oligonucleotide primers (Tsingke Biotechnology) and verified by sequencing. Electrophysiological measurements were typically performed at room temperature 24 to 48 h after transfection.

DmoGr43a currents were recorded with the whole-cell patch clamp method using an EPC10 USB amplifier (HEKA Elektronik) at a sampling rate of 10 kHz filtered at 2 kHz. The patch pipette electrodes were made from borosilicate glass using a micropipette puller (P-97 model, Sutter Instrument). The resistance of patch pipette electrodes was approximately 3–5 MΩ. The pipette solution contained (in mM): 150 KCl, 5 EGTA, 10 HEPES, pH 7.4 with N-methyl-D-glucamine (NMG). The bath solution contained (in mM): 150 NaCl, 1 $MgCl_2$, 2 $CaCl_2$, 10 HEPES, pH 7.4 with N-methyl-D-glucamine (NMG). After establishment of the whole-cell configuration, currents were measured at a holding potential of −60 mV. Fructose solution was applied via a gravity perfusion system, and fructose concentrations between 10 mM and 30 mM were used for concentration–response experiments.

Calcium imaging

The cells employed for Ca^{2+} imaging underwent co-transfection with GCaMP8f and Gr43a/Gr64a in a 1:1 ratio (2 μg +2 μg) using GenJet II. The transfected cells were transferred into an external solution and allowed to acclimate for 15 minutes prior to the commencement of imaging. The bath solution contained (in mM): 150 NaCl, 1 $MgCl_2$, 2 $CaCl_2$, 10 HEPES, pH 7.4 with N-methyl-D-glucamine (NMG).

The calcium imaging procedure was conducted using a fluorescent inverted microscope (T*i*2U, Nikon). For the capture of fluorescent signals induced by diverse sugar

compounds, the sCOMS camera (PCO. edge 4.2, PCO) was employed. Imaging was conducted at a frequency of 2 Hz, and the Nikon NIS element software was applied for the recording and processing of the acquired images.

For each given concentration of sugars, images of 20 to 60 cells were collected from 2 to 3 independent measurement areas. The fluorescence changes in these cells were grouped and used to quantify the responses of DmoGr43a/DmGr64a to sugar stimulation at each concentration. The EC_{50} values were determined by fitting the responses across multiple sugar concentrations (0.1 to 100 mM) for each mutation.

Confocal imaging and western blotting

To determine the expression level of DmoGr43a and DmGr64a variants, 1 dish of cells for each construct were cultured in the same way as for electrophysiology recordings or calcium imaging. Following extraction of proteins from pelleted cells with 1% DDM and centrifugation (14,000 RPM), total extract and supernatant were subjected to western blot analysis using antibodies against GFP (Abcam) and GAPDH (BioSS), which served as internal standard. For protein localization, 0.5 μg of plasmid encoding plasma membrane marker KRas GTPase in fusion with mCherry was co-expressed with DmoGr43a or DmGr64a variants. Differential interference contrast (DIC) and fluorescence images were taken by an inverted confocal microscope (LSM880; Carl Zeiss) using a 63×/1.46-NA oil objective lens at 20˚C. Images were processed and analyzed with ZEN 2 blue software (Carl Zeiss). Wavelengths for excitation of GFP and mCherry were 488 and 561 nm, respectively, and those for emission of GFP and mCherry were 470-510 and 560-600 nm, respectively.

All-atom molecular dynamic simulations

The tetrameric fructose-bound DmoGr43a and sucrose-bound DmGr64a was used for MD simulations. The sugar-bound DmoGr43a/DmGr64a structures with water molecules were then placed into ~13 × 13 × 15 nm (or greater) simulation boxes with CHARMM-GUI [12]. In all systems, the lipid compositions of the extracellular and intercellular leaflets were 1-palmitoyl-2-oleoyl-glycero-3-phosphocholine. Each simulation system contained 150 mM NaCl. Altogether, a typical system contained at

least ~155,000 atoms, including ~157 lipids, ~217 ions and ~40,000 water molecules (TIP3).

All simulations were performed under periodic boundary conditions using CUDA-accelerated NAMD2/3[13] and CHARMM36m force field parameters for protein and lipid[14]. During the initial equilibration, the protein's backbone atoms were harmonically restrained to their initial positions with a force constant of $k = 1$ kcal mol^{-1} Å^{-2}. The restraints were released at the start of the production run. All the non-bonded forces were calculated with a cutoff of 12 Å and a switching distance of 10 Å. Long-range electrostatic forces were calculated using the particle mesh Ewald method. A Langevin thermostat using $\gamma = 1$ ps^{-1} was used to maintain the system temperature at 303.5 K. The pressure was maintained at 1 bar using a Nosé–Hoover–Langevin piston method. An integration time step of 2 fs was used in all the simulations.

The r.m.s.d values were relatively stable after ~30 ns of simulation. To characterize sugars–protein interaction sites, the residues where the heavy atoms are located within 4 Å around fructose or sucrose are calculated using VMD statistics, and the probabilities are normalized to 1 for all frames that are in contact. The simulation results were analyzed in VMD (v1.9; http://www.ks.uiuc.edu/), PyMol (v1.8 and v2.0; Schrödinger) [10] and Igor Pro (WaveMetrics).

References

1 Morales-Perez, C. L., Noviello, C. M. & Hibbs, R. E. Manipulation of Subunit Stoichiometry in Heteromeric Membrane Proteins. *Structure* **24**, 797-805, doi:10.1016/j.str.2016.03.004 (2016).

2 Gao, Y., Cao, E., Julius, D. & Cheng, Y. TRPV1 structures in nanodiscs reveal mechanisms of ligand and lipid action. *Nature* **534**, 347-351, doi:10.1038/nature17964 (2016).

3 Zheng, S. Q. *et al.* MotionCor2: anisotropic correction of beam-induced motion for improved cryo-electron microscopy. *Nat Methods* **14**, 331-332, doi:10.1038/nmeth.4193 (2017).

4 Scheres, S. H. RELION: implementation of a Bayesian approach to cryo-EM structure determination. *J Struct Biol* **180**, 519-530, doi:10.1016/j.jsb.2012.09.006 (2012).

5 Punjani, A., Rubinstein, J. L., Fleet, D. J. & Brubaker, M. A. cryoSPARC: algorithms for rapid unsupervised cryo-EM structure determination. *Nat Methods* **14**, 290-296, doi:10.1038/nmeth.4169 (2017).

6 Adams, P. D. *et al.* PHENIX: a comprehensive Python-based system for macromolecular structure solution. *Acta Crystallogr D Biol Crystallogr* **66**, 213-221,

doi:10.1107/S0907444909052925 (2010).
7 Chen, V. B. *et al.* MolProbity: all-atom structure validation for macromolecular crystallography. *Acta Crystallogr D Biol Crystallogr* **66**, 12-21, doi:10.1107/S0907444909042073 (2010).
8 Smart, O. S., Neduvelil, J. G., Wang, X., Wallace, B. A. & Sansom, M. S. HOLE: a program for the analysis of the pore dimensions of ion channel structural models. *J Mol Graph* **14**, 354-360, 376 (1996).
9 Wallace, A. C., Laskowski, R. A. & Thornton, J. M. LIGPLOT: a program to generate schematic diagrams of protein-ligand interactions. *Protein Eng* **8**, 127-134, doi:10.1093/protein/8.2.127 (1995).
10 Schrodinger, LLC. *The PyMOL Molecular Graphics System, Version 1.8* (2015).
11 Pettersen, E. F. *et al.* UCSF ChimeraX: Structure visualization for researchers, educators, and developers. *Protein Sci* **30**, 70-82, doi:10.1002/pro.3943 (2021).
12 Jo, S., Kim, T., Iyer, V. G. & Im, W. CHARMM-GUI: a web-based graphical user interface for CHARMM. *J Comput Chem* **29**, 1859-1865, doi:10.1002/jcc.20945 (2008).
13 Phillips, J. C. *et al.* Scalable molecular dynamics with NAMD. *J Comput Chem* **26**, 1781-1802, doi:10.1002/jcc.20289 (2005).
14 Klauda, J. B. *et al.* Update of the CHARMM all-atom additive force field for lipids: validation on six lipid types. *J Phys Chem B* **114**, 7830-7843, doi:10.1021/jp101759q (2010).

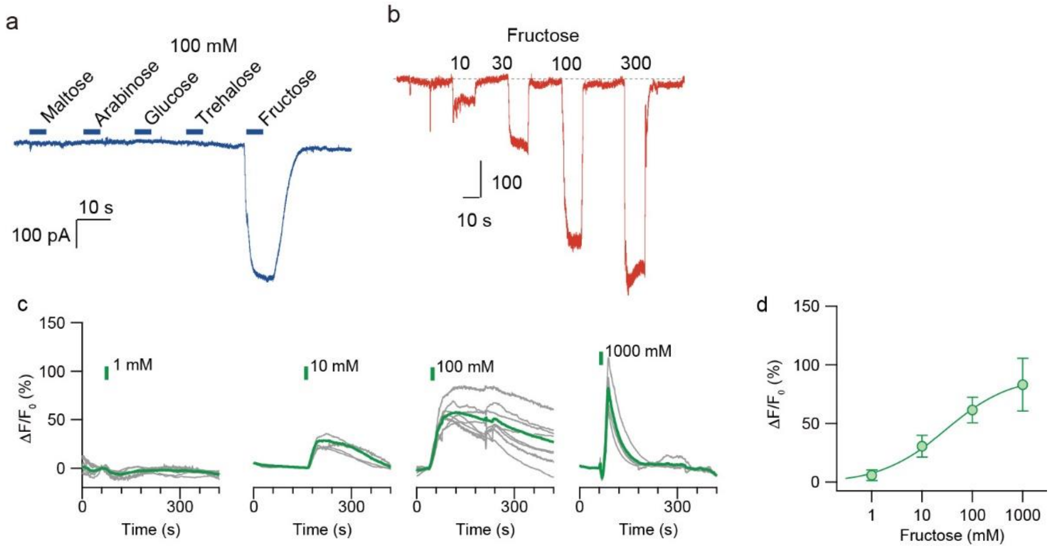

Supplementary Fig. S1. Functional characterization of DmoGr43a by electrophysiology and fluorescence measurements. a Sample current traces of DmoGr43a-expressing HEK293 cells, in which different sugars were applied sequentially, as indicated above. **b** Sample current traces of DmoGr43a-expressing HEK293 cells, in which increasing concentration (in mM) of fructose were applied sequentially, as indicated above. **c-d** Fluorescence intensity changes induced by fructose (**c**) and the resulting concentration-response relationship (**d**) of DmoGr43a-expressing HEK293 cells, in which increasing concentration (in mM) of fructose were applied as indicated above.

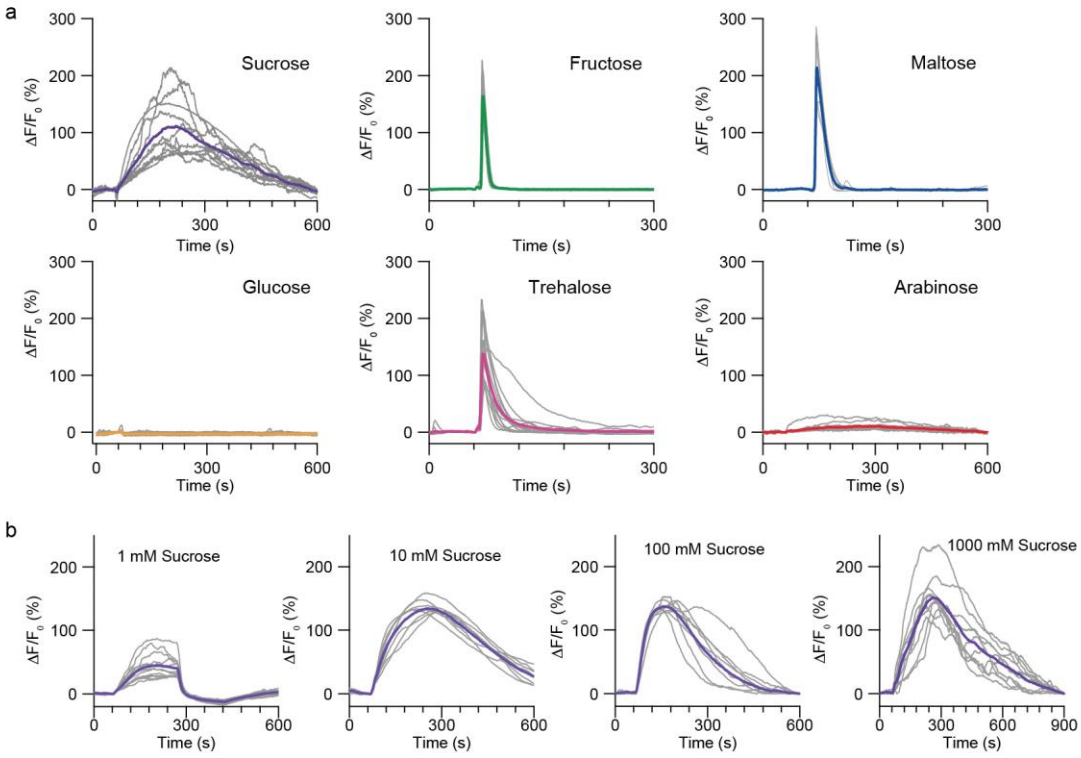

Supplementary Fig. S2. Functional characterization of DmGr64a by fluorescence measurements. a Fluorescence intensity changes of DmGr64a-expressing HEK293 cells induced by various sugars. In each panel, various sugars (100 mM) were applied as indicated. **b** Fluorescence intensity changes of DmGr64a-expressing HEK293 cells induced by sucrose. Increasing concentration (in mM) of sucrose were applied as indicated above.

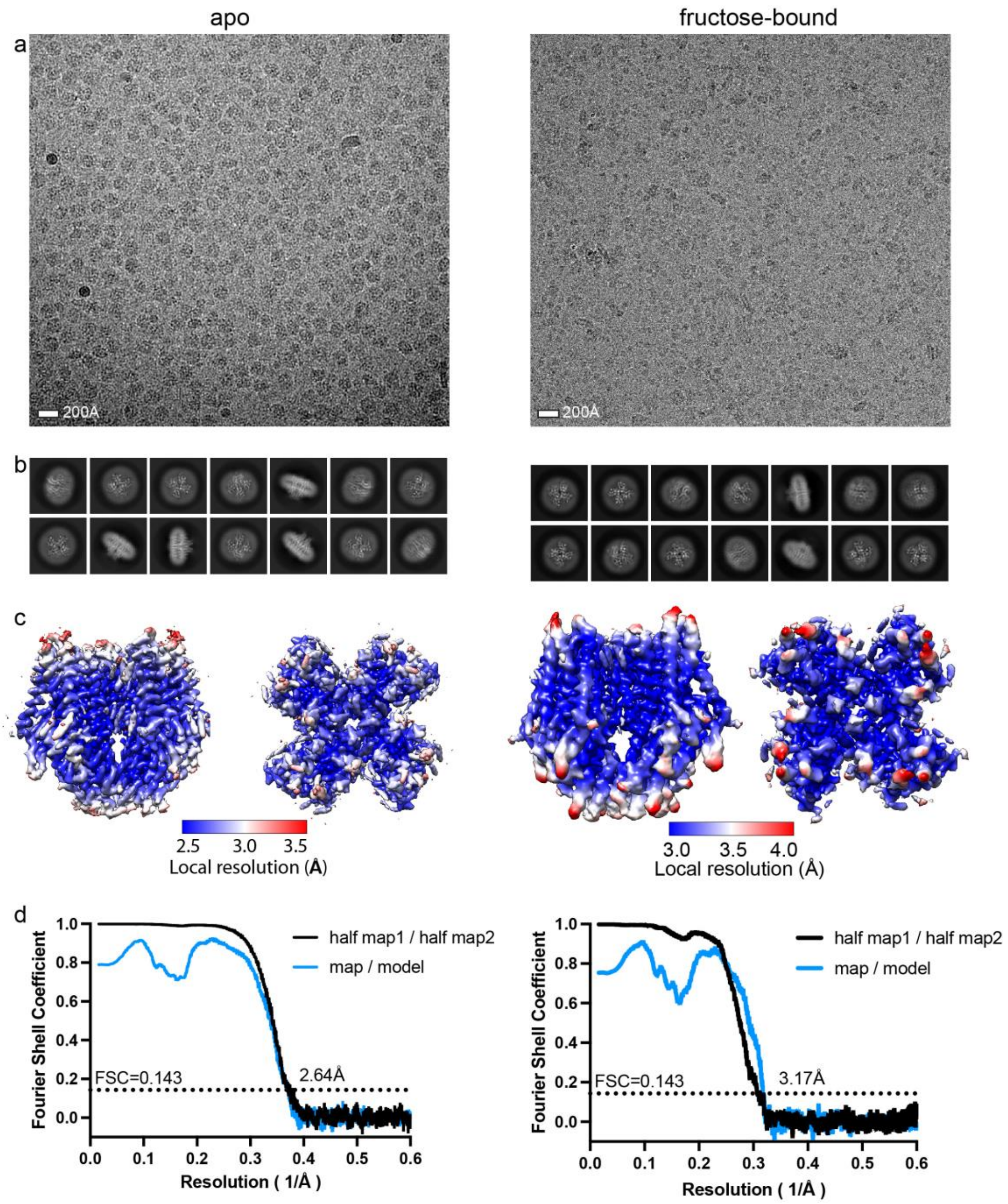

Supplementary Fig. S3. Cryo-EM analysis of DmoGr43a in apo and fructose-bound states. a A representative cryo-EM micrograph of DmoGr43a. Scale bar, 200 Å. **b** 2D class average images of DmoGr43a. **c** Local resolution distribution for the density map of DmoGr43a. **d** The GSFSC curve for the reconstruction of DmoGr43a.

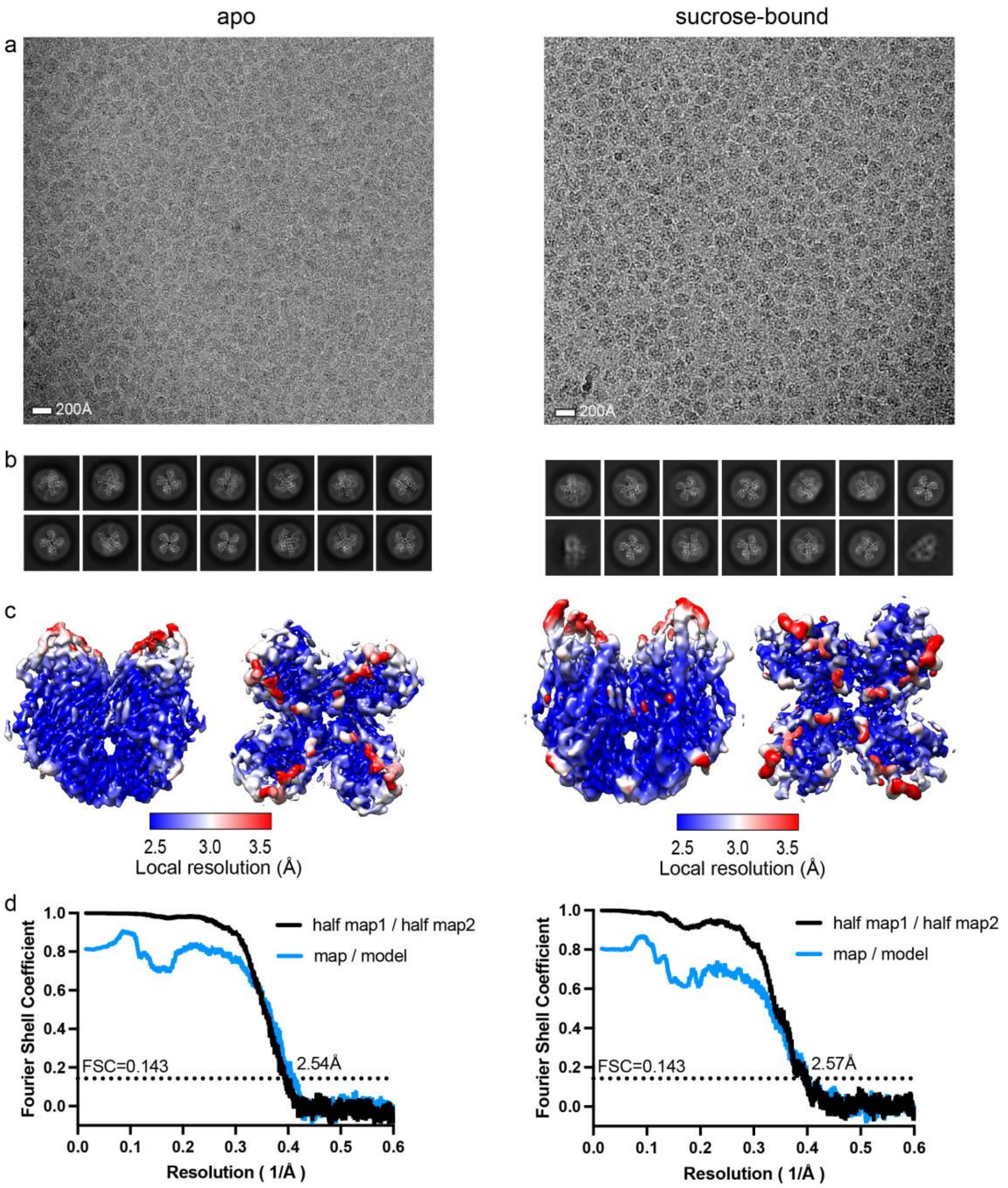

Supplementary Fig. S4. Cryo-EM analysis of DmGr64a in apo and sucrose-bound states. a A representative cryo-EM micrograph of DmGr64a. Scale bar, 200 Å. **b** 2D class average images of DmGr64a. **c** Local resolution distribution for the density map of DmGr64a. **d** The GSFSC curve for the reconstruction of DmGr64a.

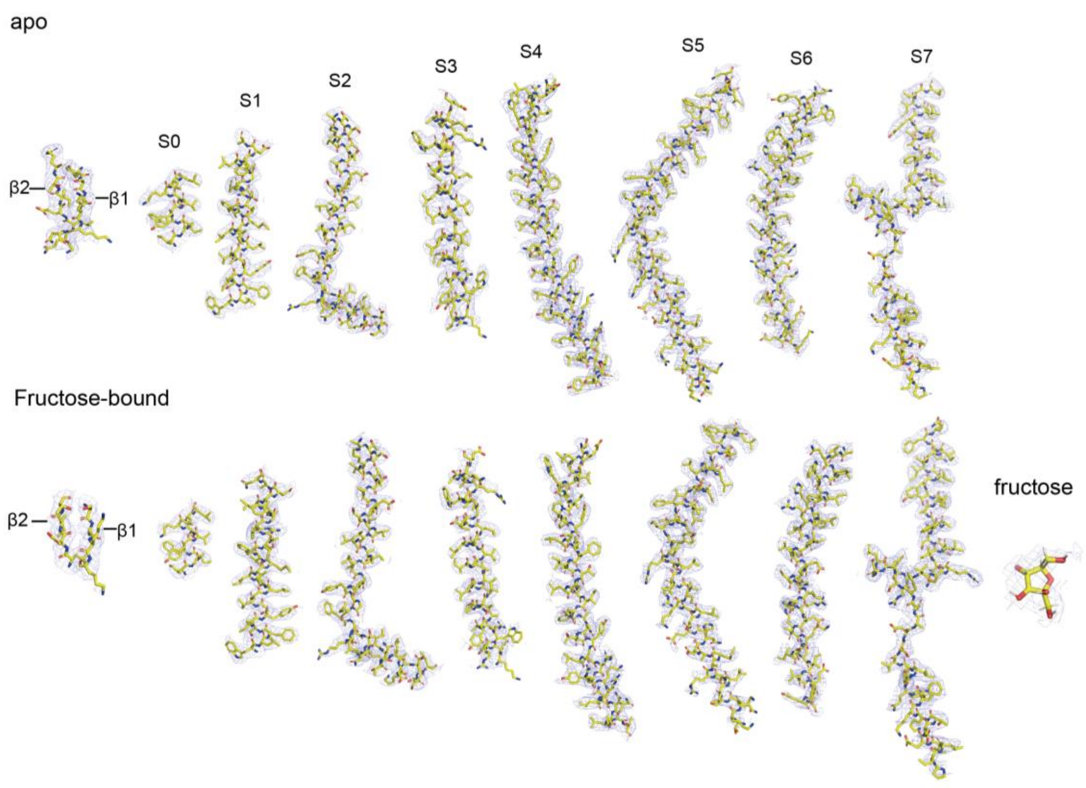

Supplementary Fig. S5. Sample electron microscopy densities for various parts of DmoGr43a apo and fructose-bound structures. The maps are low-pass filtered to 3Å and sharpened with a temperature factor of −100 $Å^2$.

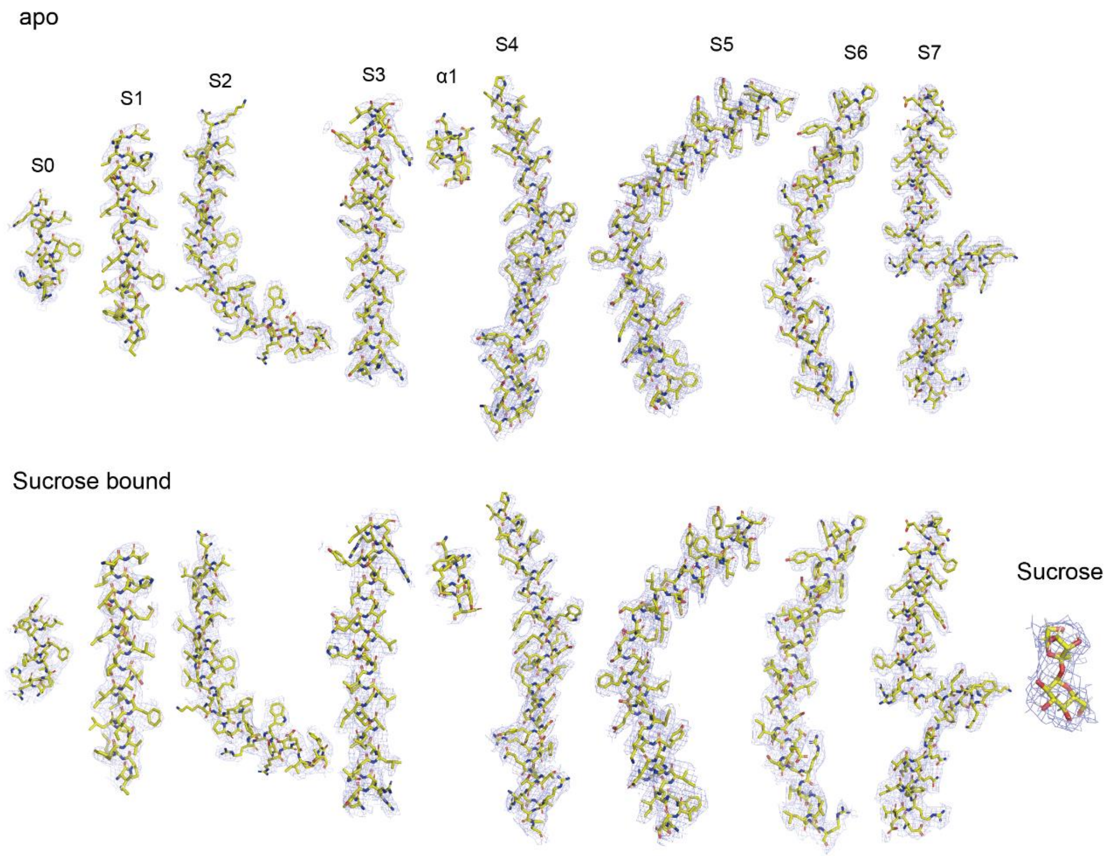

Supplementary Fig. S6. Sample electron microscopy densities for various parts of DmGr64a apo and sucrose-bound structures. The maps are low-pass filtered to 3Å and sharpened with a temperature factor of −100 Å^2.

a

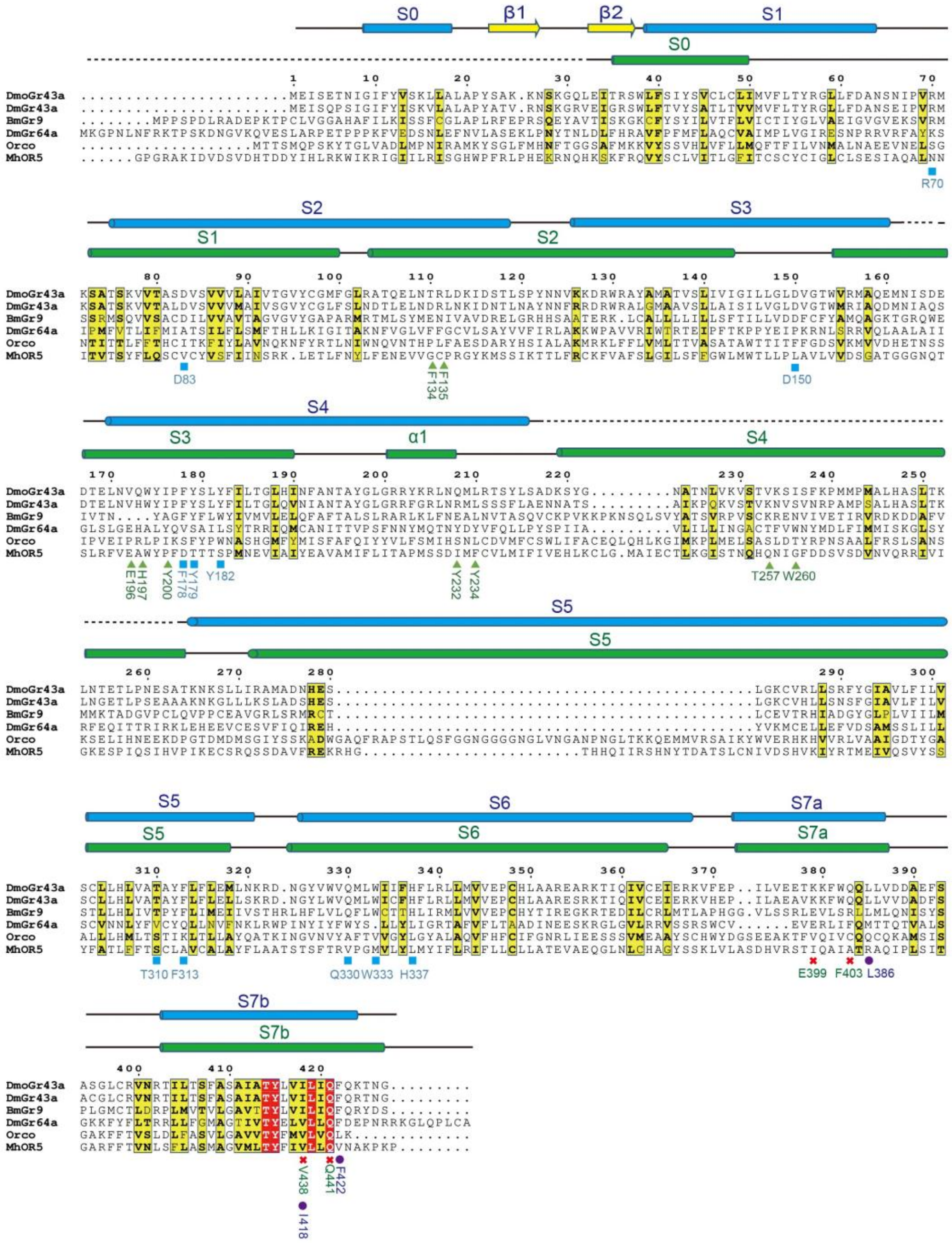

b

DmoGr43a	100%					
DmGr43a	73.77%	100%				
BmGr9	24.59%	24.12%	100%			
DmGr64a	12.41%	11.47%	8.68%	100%		
Orco	10.77%	10.30%	10.46%	10.96%	100%	
MhOR5	9.36%	9.36%	6.68%	10.30%	14.01%	100%
	DmoGr43a	DmGr43a	BmGr9	DmGr64a	Orco	MhOR5

Sequence identity

DmoGr43a	100%					
DmGr43a	80.09%	100%				
BmGr9	36.76%	35.83%	100%			
DmGr64a	23.65%	23.18%	19.37%	100%		
Orco	19.43%	19.43%	19.59%	20.39%	100%	
MhOR5	18.96%	19.67%	18.04%	22.36%	25.73%	100%
	DmoGr43a	DmGr43a	BmGr9	DmGr64a	Orco	MhOR5

Sequence similarity

Supplementary Fig. S7. Sequence alignment of DmoGr43a, DmGr43a, BmGr9, DmGr64a, Orco and MhOR5 (a) and their sequence identity and similarity (b). a Sequence alignment of *Drosophila mojavensis* Gr43a (accession number EDW09995.2), *Drosophila melanogaster* Gr43a (accession number Q9V4K2.3), *Bombyx mori* Gr9 (accession number NP_001124345.1), *Drosophila melanogaster*

Gr64a (accession number NP_728920.1), *Apocrypta bakeri* Orco (accession number NP_001097687.1), *Machilis hrabei* OR5 (accession number 7LIG_D). Secondary structures are colored as in Fig. 2 and are indicated by cylinders (helices), arrows (β-strands), solid line (loop), and dashed line (disordered region). Residues involved in fructose binding in DmoGr43a are indicated using green triangles, whereas those involved in sucrose binding in DmGr64a are indicated using blue squares. In the pore, residues forming constrictions in DmoGr43a are indicated using purple circles, whereas those forming constrictions in DmGr64a are indicated using red crosses. **b** Tables showing the sequence identity and similarity among proteins used for sequence alignment in **a**.

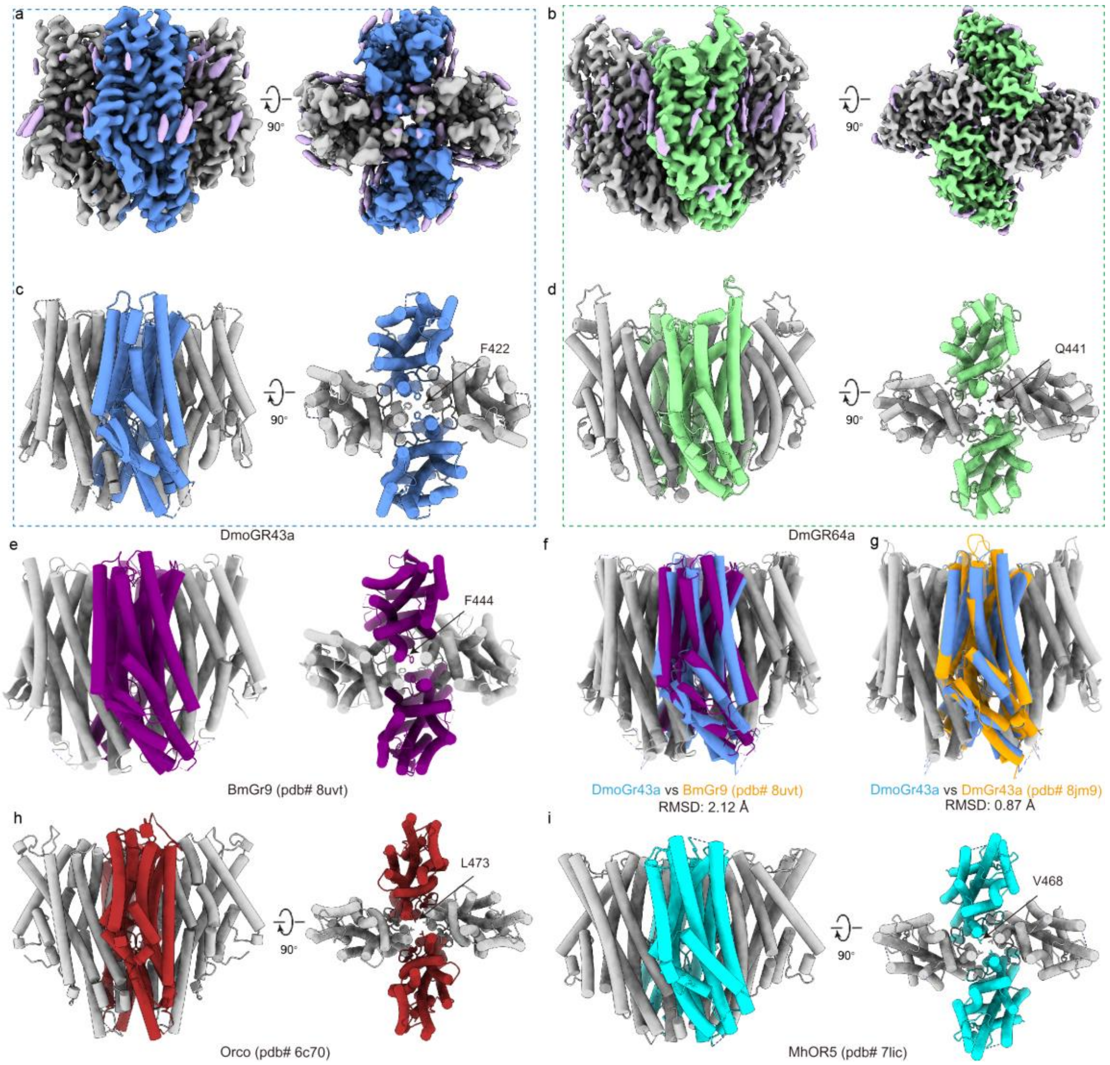

Supplementary Fig. S8. Structures of DmoGr43a, DmGr64a and its comparison with structures of other Grs, Orco and MhOR5. a-b, Electron density maps of DmoGr43a (**a**) and DmGr64a (**b**) in apo state. **c-e**, Cartoon representations of DmoGr43a (**c**), DmGr64a (**d**), and BmGr9 (**e**) structures in apo state. **f-g**, Superimposition of DmoGr43a structure with that of BmGr9 (**f**) and DmGr43a (**g**). **h-i,** Cartoon representations of Orco (**h**) and MhOR5 (**i**) structures in apo state. Main chains shown as cartoons, and residues forming constrictions at extracellular entrance shown as sticks and labeled. Two views are shown in all panel except **f** and **g**.

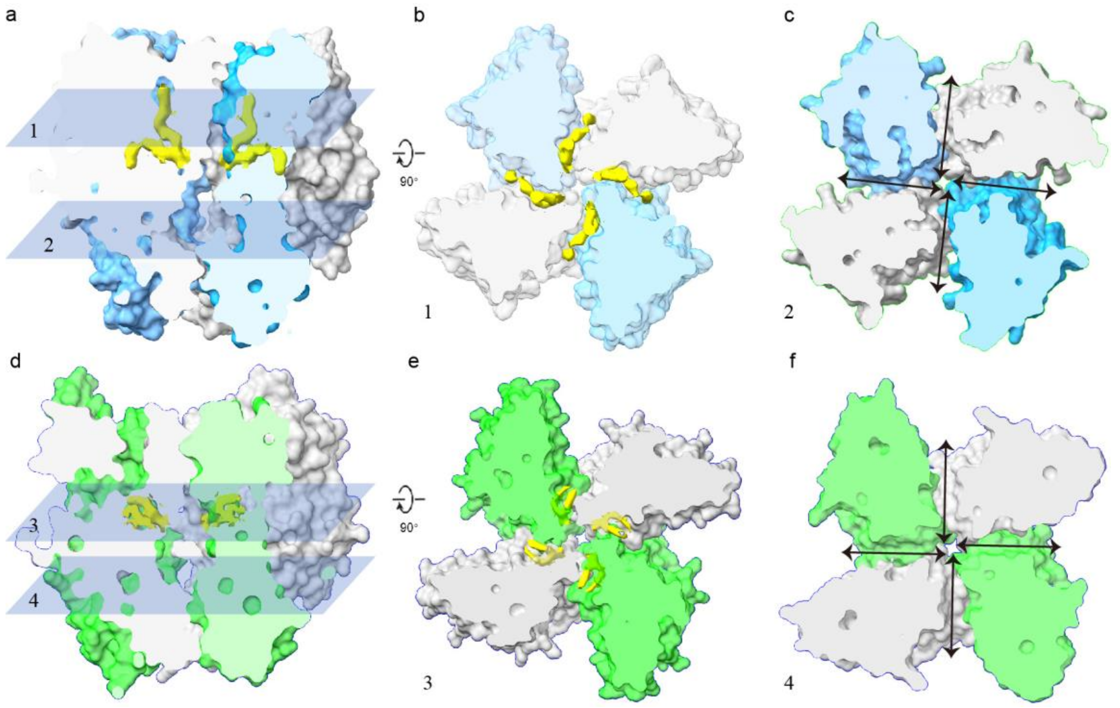

Supplementary Fig. S9. Comparison of sugar bound structures of DmoGr43a (a-c), and DmGr64a (d-f), highlighting their bound lipids (a-b&d-e) and the side fenestrations (c&f) at the ICD-TMD junction. Structures are shown as surface representations. Densities for bound lipids are shown as surfaces and colored yellow. In all panels, structures are sliced for clarity. Positions of slicing are indicated and numbered accordingly. In **c&f**, arrows indicate the potential ion passage pathways.

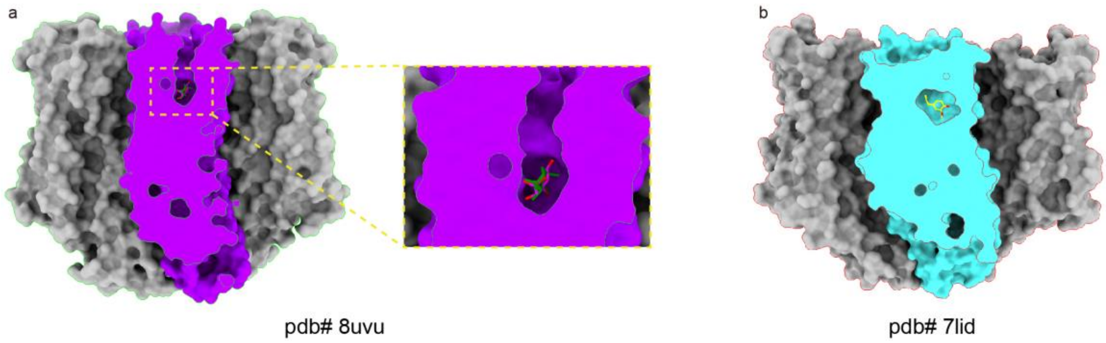

Supplementary Fig. S10. Ligand binding pocket of BmGr9 and MhOR5. Surface representation of fructose-bound BmGr9 structure (**a**) and eugenol-bound MhOR5 structure (**b**) in front view, with the foremost subunit sliced in order to clearly show the ligand binding site. In **a**, inset shows an enlarged view of the fructose binding pocket, with beta-D-fructopyranose and beta-D-fructofuranose shown as magenta and green sticks respectively.

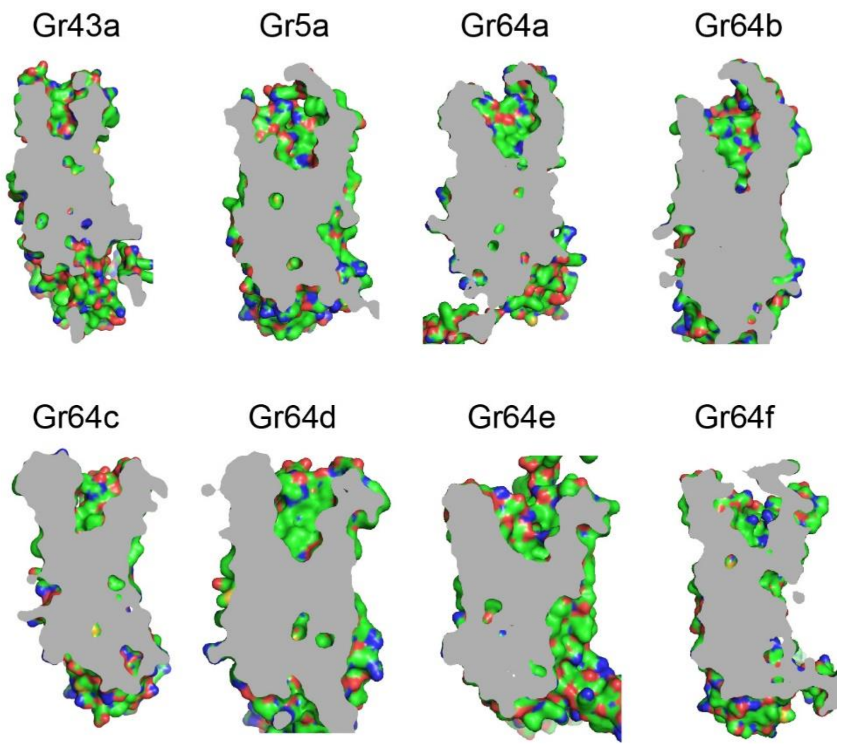

Supplementary Fig. S11. Comparison of AlphaFold models of DmoGr43a, DmGr5a, DmGr64a-f, highlighting the size of sugar binding pocket. Single subunit AlphaFold models of DmGr5a (AF-Q9W497-F1), DmoGr43a (AF-Q9V4K2-F1), DmGr64a (AF-P83293-F1), DmGr64b (AF-P83294-F1), DmGr64c (AF-P83295-F1), DmGr64d (AF-Q9VZJ6-F1), DmGr64e (AF-P83296-F1), and DmGr64f (AF-P83297-F1) are shown as surface representations. For DmGr64c, DmGr64d and DmGr64f, the C-termini were removed, as these regions are predicted to insert into the sugar binding pocket and clog the binding pocket, which is rather unlikely.

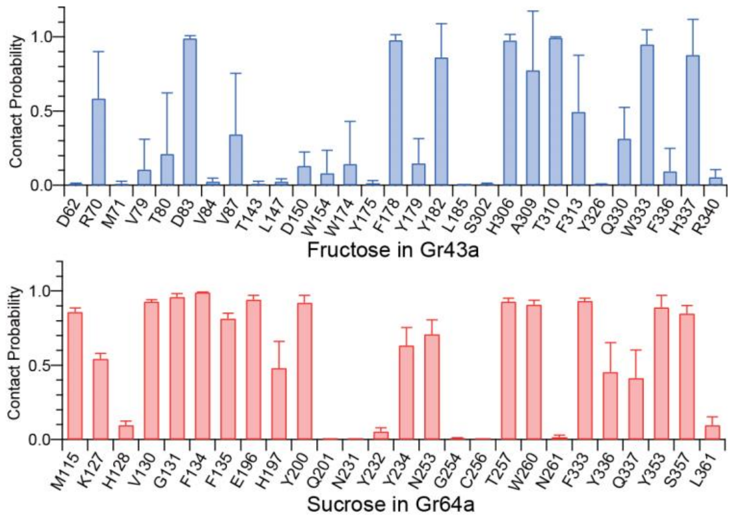

Supplementary Fig. S12. The contact probability for fructose and sucrose with key residues in the binding pocket of Gr43a and Gr64a during MD simulation. A heavy-atom distance cutoff of 4 Å was used to define a contact between a residue and sugar molecules. Data represent mean ± SD for 4 trajectories within a tetrameric channel.

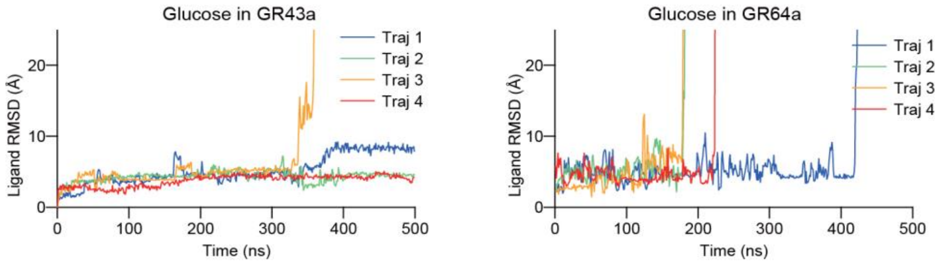

Supplementary Fig. S13. MD simulations of glucose modeled in the sugar binding pocket of Gr43a and Gr64a. For each model, the time-dependent RMSD of glucose with respect to their starting coordinates during the 500-ns simulation is shown. RMSDs of 4 bound glucoses in each tetrameric channel are overlaid. Note that while only one glucose has left the binding pocket in Gr43a at later stage of the simulation, all 4 glucoses have left the binding pocket in Gr64a at different time points during the simulation.

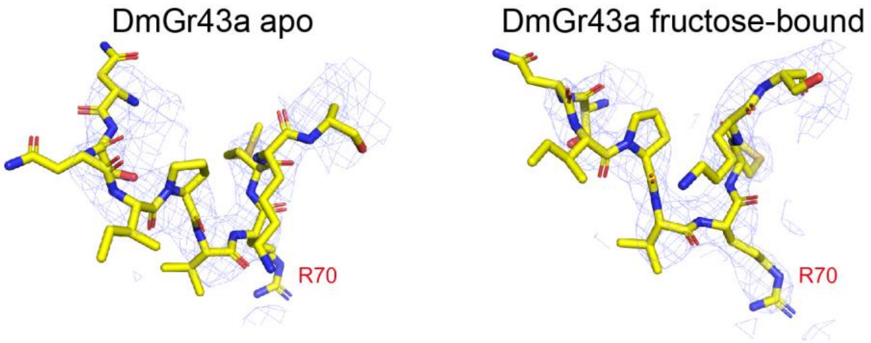

Supplementary Fig. S14. Comparison of electron densities of the S1-S2 loop between apo and fructose-bound DmoGr43a structures, highlighting the transition of Arg 70 side chain from being disordered to ordered. Polypeptide is shown as yellow sticks, and electron densities are shown as blue mesh.

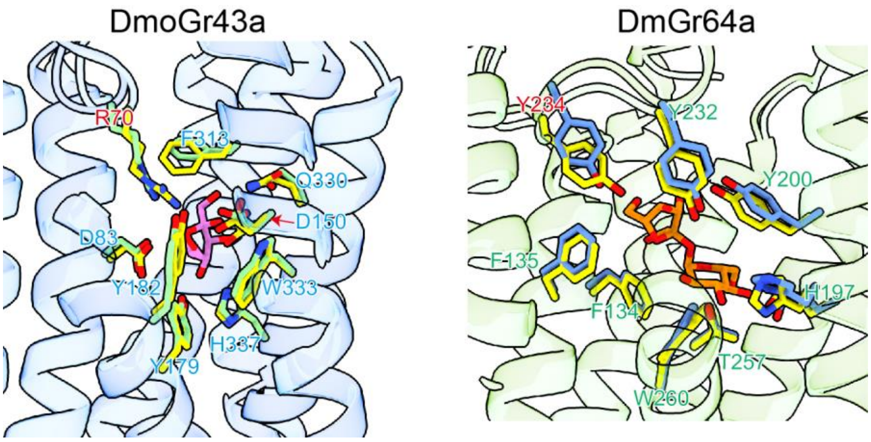

Supplementary Fig. S15. Conformational changes around the sugar binding pocket of DmoGr43a and DmGr64a. Structures are shown as cartoons, whereas fructose and sucrose molecules are shown as violet and orange sticks. For DmoGr43a, residues in apo state are shown as green sticks and those in fructose-bound state are shown as yellow sticks. For DmGr64a, residues in apo state are shown as blue sticks and those in sucrose-bound state are shown as yellow sticks. All residues are labeled. Arg 70 in DmoGr43a and Tyr 234 in DmGr64a, which are both located in an extracellular loop and undergo dramatic conformational changes, are labeled in red.

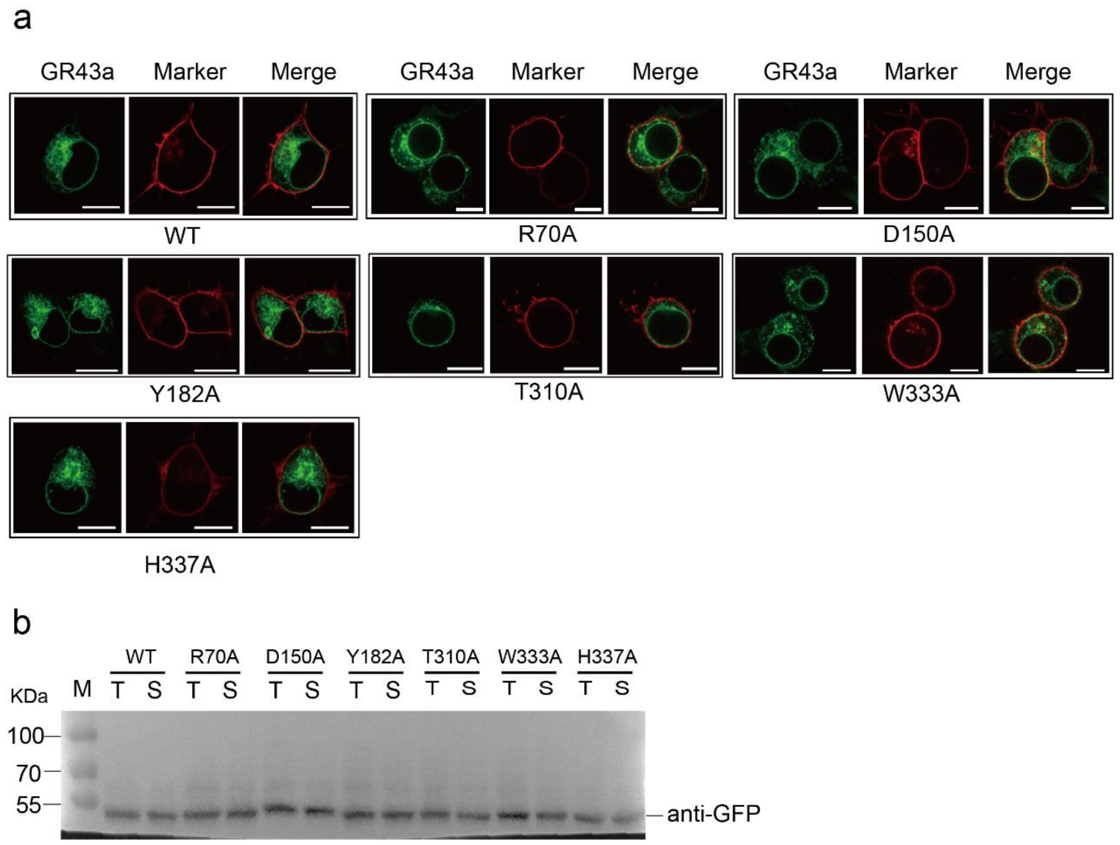

Supplementary Fig. S16. Expression and subcellular localization of DmoGr43a. a, Subcellular localization as determined by confocal imaging. From left to right, three images in each box represent the indicated DmoGr43a variant fused with GFP, membrane marker KRas GTPase fused with mCherry, and merge, respectively. The scale bars represent 5 µm. The experiment was repeated twice independently with similar results. **b**, Protein expression level as determined by Western blotting. T: total extract after detergent solubilization. S: supernatant after centrifugation. The experiment was repeated twice independently with similar results.

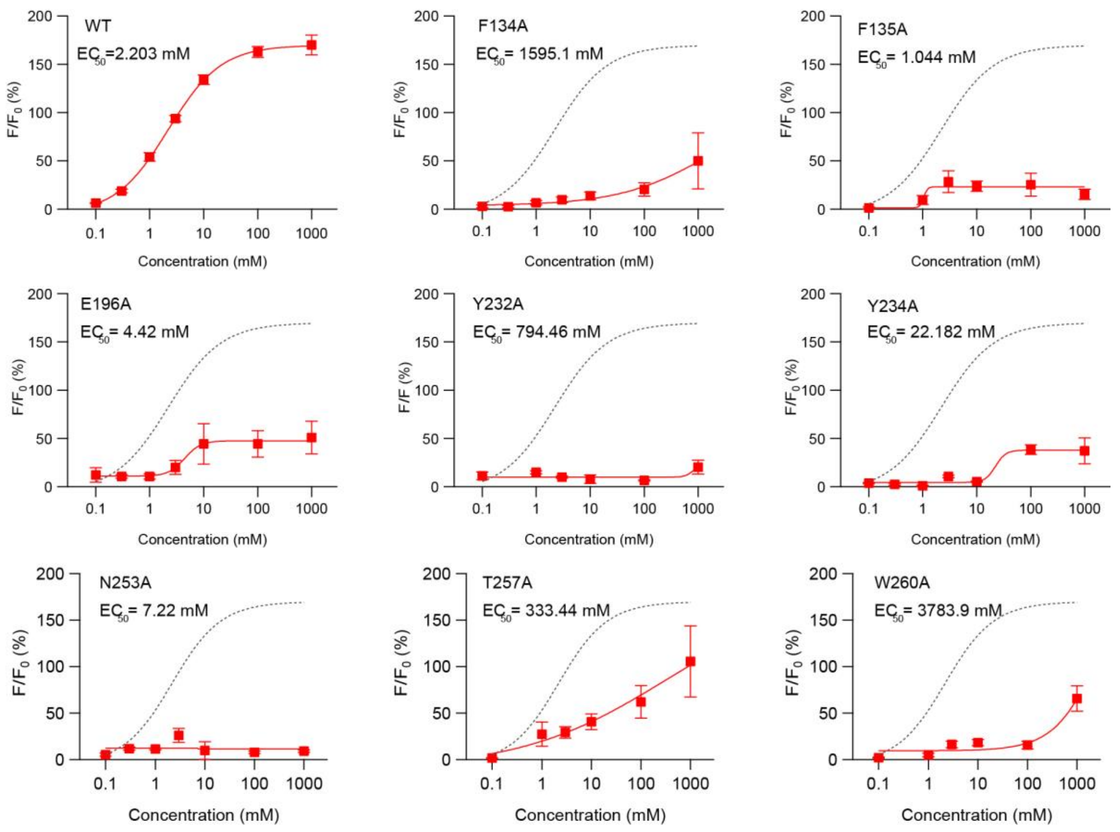

Supplementary Fig. S17. Concentration-response curves of WT and sucrose binding residues mutants of Dm64a resulted from Ca^{2+} imaging. Increasing concentration of sucrose was applied by perfusion and the fluorescence signal were quantified and used for generation of concentration-response curves. In each panel, the curve for each indicated mutant is shown as a solid line and is overlaid with that of WT (dashed line). All data points are mean ± SEM.

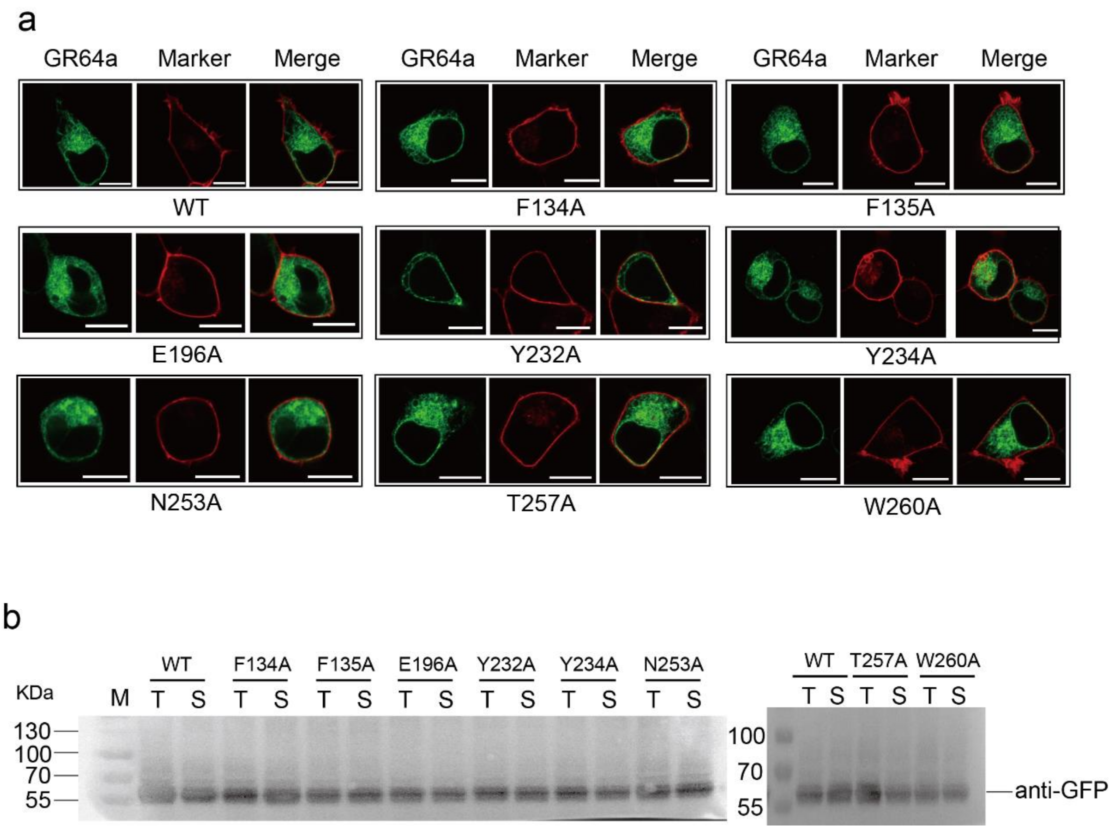

Supplementary Fig. S18. Expression and subcellular localization of DmGr64a. a, Subcellular localization as determined by confocal imaging. From left to right, three images in each box represent the indicated DmGr64a variant fused with GFP, membrane marker KRas GTPase fused with mCherry, and merge, respectively. The scale bars represent 5 μm. The experiment was repeated twice independently with similar results. **b**, Protein expression level as determined by Western blotting. T: total extract after detergent solubilization. S: supernatant after centrifugation. The experiment was repeated twice independently with similar results.

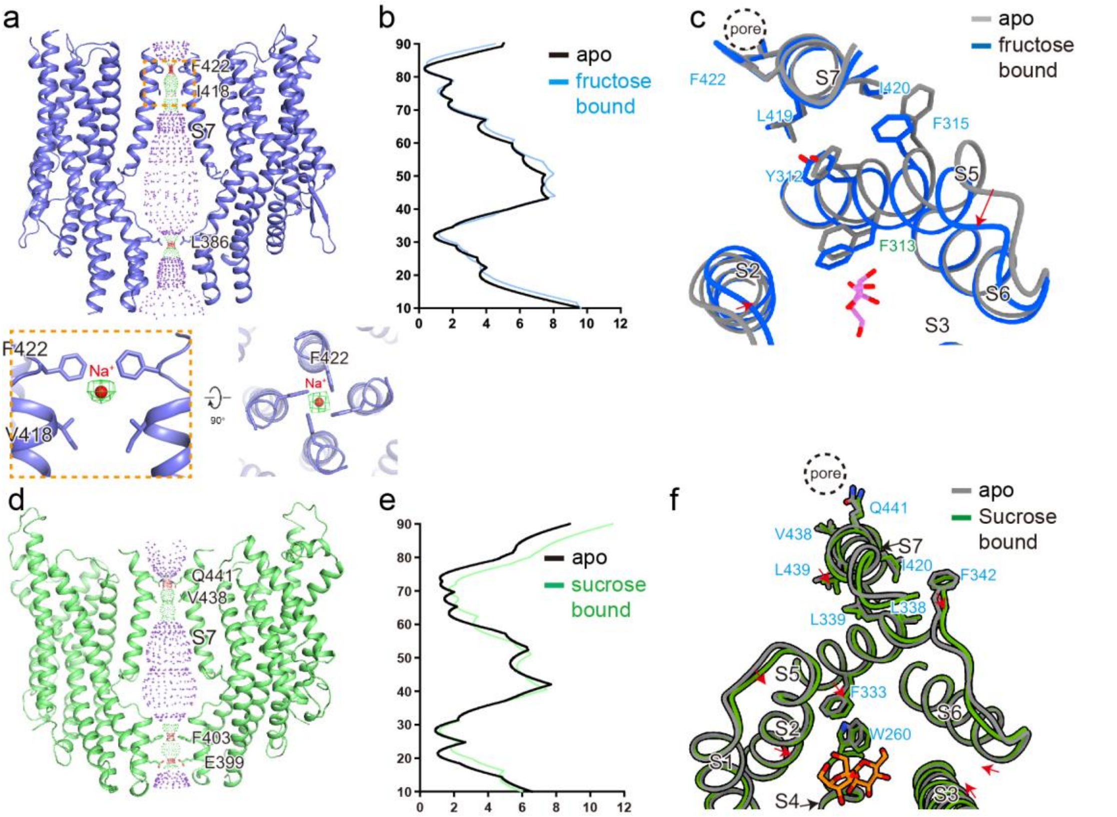

Supplementary Fig. S19. Ion conduction pathway and propagation of conformational changes towards ion conduction pore in DmoGr43a and DmGr64a. Ion conduction pathway and pore radius of DmoGr43a (**a&b**) and DmGr64a (**d&e**) structures calculated by HOLE program. Residues forming constrictions are shown as sticks. In **a**, the inset at the bottom shows expanded views (area indicated in the dashed box) of the density for the modeled Na^+, which is the predominant ion specie in the purification buffer. **c&f**, Superimposition of single subunit of apo and sugar-bound DmoGr43a and DmGr64a structures, highlighting the structural changes upon fructose binding. Key residues likely for channel activation in apo and sugar-bound DmoGr43a and DmGr64a structures are shown as sticks, and color coded similarly as in **b&e**. Fructose and sucrose molecules are shown as sticks, and colored violet and orange respectively.

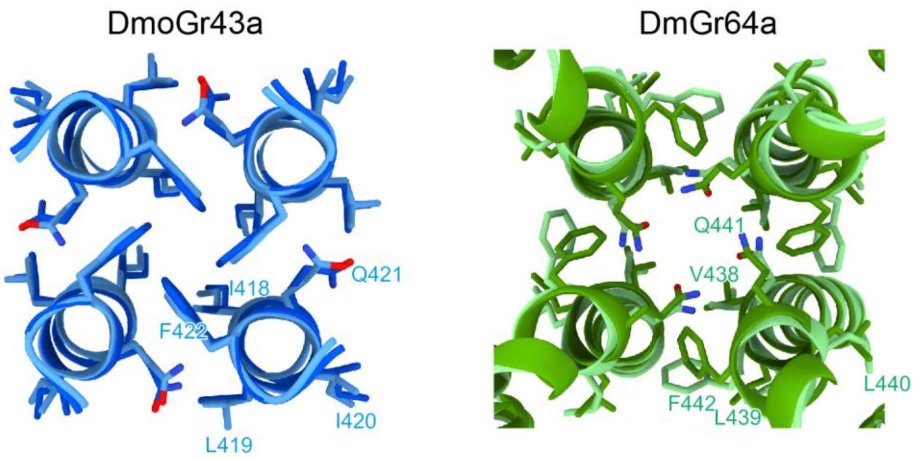

Supplementary Fig. S20. Differences in residues around constrictions at extracellular entrance of DmoGr43a and DmGr64a. S7s are shown as cartoons, and key residues are shown as sticks and labeled for one subunit. Apo and fructose-bound structures of DmoGr43a are colored in dark and pale blue, whereas apo and sucrose-bound structures of DmGr64a are colored in dark and pale green.

Supplementary Table S1. Statistics for cryo-EM data collection

	apo Gr43a	Gr43a-Fructose (100 mM)	apo Gr64a	Gr64a-sucrose (50 mM)
**Data collection and processing**				
Total movies #	7,587	7,965	3,342	4,005
Magnification	36,000 x	36,000 x	36,000 x	36,000 x
Voltage (KV)	300	300	300	300
Electron exposure ($e^-/Å^2$)	49.95 $e^-/Å$	48.66 $e^-/Å$	49.52 $e^-/Å$	48.24 $e^-/Å$
Defocus range (um)	-0.8 to -1.8	-0.8 to -1.8	-0.8 to -1.8	-0.8 to -1.8
Super resolution pixel size ($Å^2$)	0.466	0.466	0.466	0.466
Binned pixel size ($Å^2$)	0.932	0.932	0.932	0.932
Final particle images (no.)	485,547	82,442	317,046	413,565
Map resolution	2.64	3.17	2.54	2.57
Symmetry	C4	C4	C4	C4
**Refinement**				
Model resolution (Å, FSC = 0.143 / FSC = 0.5)	2.7/3.0	3.1/3.4	2.5/2.8	2.5/3.2
Map-sharpening B factor ($Å^2$)	100	150	80	100
Composition				
Number of atoms	12024	12120	12880	13060
Number of protein residues	1496	1496	1568	1568
Ligand		4		4
R.m.s. deviations				
Bond lengths (Å)	0.003	0.002	0.003	0.003
Bond angles (Å)	0.629	0.364	0.596	0.543
Validation				
MolProbity score	1.75	1.92	1.51	2.53
Clash score	6.61	26.95	7.98	52.37

Ramachandran plot				
Favored (%)	97.28	98.17	997.63	95.13
Allowed (%)	2.72	1.83	2.37	4.87
Disallowed (%)	0	0	0	0
Rotamer outliers (%)	2.12	0.15	0.00	0.00
Mean B factor ($Å^2$)				
Protein	117.93	118.19	83.96	85.24
Ligand	-	111.69	-	98.37
Deposited model	8ZDZ	8ZE3	8ZE0	8ZE2
Deposited map	EMD-60018	EMD-60022	EMD-60019	EMD-60021
